# Assessing Health-Related Quality of Life in Mycosis Fungoides and Sézary Syndrome: Unmet Needs

**DOI:** 10.3390/cancers16152757

**Published:** 2024-08-03

**Authors:** Danielle Brazel, Cecilia Larocca, Michi M. Shinohara

**Affiliations:** 1 Department of Hematology/Oncology, Scripps Clinic/Scripps Green Hospital, La Jolla, CA 92037, USA; 2 Brigham and Women’s Department of Dermatology, Dana Farber Cancer Center, Boston, MA 02215, USA; 3 Department of Dermatology, University of Washington, 1959 Northeast Pacific Street BB-1353, Box 356524, Seattle, WA 98195, USA

**Keywords:** health related quality of life, mycosis fungoides, Sézary syndrome

## Abstract

**Simple Summary:**

Mycosis fungoides and Sézary syndrome can impair multiple dimensions of health-related quality of life due to physical symptoms including pruritus, treatment side effects, time and financial burdens associated with high healthcare utilization, and psychological distress. There are several areas of unmet needs around health-related quality of life in this population, and the optimal way to measure health-related quality of life isn’t yet clear.

**Abstract:**

Mycosis fungoides (MF) and Sézary syndrome (SS) can impair multiple dimensions of health-related quality of life (HRQoL). Currently, there is no standardized assessment tool for measuring HRQoL in patients with MF/SS. Here, we describe the existing literature on multiple dimensions of HRQoL in MF/SS with a special focus on the gaps in the current knowledge and identify future directions necessary to assess the HRQoL of patients with this disease.

## 1. Introduction

Cutaneous T-cell lymphoma (CTCL) describes a heterogeneous group of non-Hodgkin lymphomas that primarily affect the skin, with mycosis fungoides (MFs) and Sézary syndrome (SS) being common subtypes. Other subtypes include CD30+ lymphoproliferative disorders such as primary cutaneous anaplastic large-cell lymphoma, lymphomatoid papulosis, and non-MF CTCLs such as adult T-cell leukemia/lymphoma, subcutaneous panniculitis like T-cell lymphoma, and primary cutaneous peripheral T-cell lymphoma. MF is a T-cell non-Hodgkin lymphoma characterized by skin lesions such as erythroderma, patches, plaques, or tumors. Skin lesions may be localized or widespread and include additional sites such as lymph nodes or viscera. SS is a distinctive erythrodermic T cell non-Hodgkin lymphoma with leukemic involvement by T cells with clonal receptor rearrangements. SS can present de novo with skin, blood, or nodal involvement or it may evolve from MF. MF and SS share many histologic and clinical features; however, patients with SS frequently are more symptomatic, have lower remission rates, and have shorter survival compared to MF patients. Many patients with MF/SS experience indolent disease, with a clinical course that can span decades [1].

Involvement of MF/SS can vary from localized patches/plaques in early stages to ulcerative tumors or erythroderma with or without extracutaneous involvement in more advanced stages [2,3,4]. Severity of disease is determined by staging, which describes lesion morphology and compartment(s) involved including skin (patch, plaque, tumor, erythroderma), blood, lymph nodes, or viscera. The staging of MF/SS is summarized in Table 1. Although the standard accepted staging criteria were established by Olsen et al., there is some subjectivity in the criteria. For example, one study found high divergence rates (25%) among experts when distinguishing between thin and thick plaques [5].

Treatment of MF/SS depends on the stage of disease. Early-stage disease can be treated with skin-directed therapy such as topical agents, phototherapy, and radiation [6]. Although skin-directed therapy is generally well-tolerated, it can be very time-consuming for patients, and can be associated with skin and hair changes in the treatment area. Generalized or advanced disease can be treated with retinoids, methotrexate, interferons, extracorporeal photopheresis, histone deacetylase inhibitors, alemtuzumab, single-agent chemotherapies or combination therapy of the above with or without skin-directed therapy. Relapsed or refractory disease can be managed with new agents such as mogamulizumab, brentuximab vedotin, or pembrolizumab. These agents have associated toxicity profiles, require regular clinic visits for monitoring, and can be expensive for the patient and/or healthcare system. Standard systemic treatments and their most common side effects are listed in Table 2 [7,8].

## 2. Quality of Life in MF/SS

Measuring patient well-being and overall health was incorporated in medical decision-making in the 1970s, particularly for survival decisions [9,10]. The Food and Drug Administration (FDA) has recognized that patient-reported outcomes, including those that relate to QoL, relief of tumor-related symptoms, and drug toxicity, should be considered in the approval process for oncology drugs [11]. Patient QoL is regularly taken into consideration for drug approval in the European Union.

MF/SS are largely incurable, though highly treatable conditions. Although many patients with MF/SS have a good prognosis, patients may live for decades experiencing significant symptoms and distress from either their disease or the associated treatments [12]. Treatment is predominantly palliative with a focus on symptom control, emphasizing the importance of measuring QOL in this patient population. 

Currently, there is no standardized assessment tool of HRQoL in patients with MF/SS, and it is unclear how the combination of dermatologic symptoms and cancer diagnosis interplay. Using a patient-reported outcome measure (PROM) to assess HRQoL can help monitor the impact of disease, measure the benefit of treatment, and prioritize symptoms to focus on during clinical encounters. Here, we review the dermatologic and oncologic tools commonly used to assess HRQoL in patients with MF/SS, with a focus on highlighting the unmet needs in the existing literature on HRQoL in MF/SS.

## 3. Dermatologic-Specific Assessments of HRQoL

Chronic skin diseases can cause significant psychological and social distress [13] and HRQoL in dermatology patients is considered a significant cause of non-fatal disease burden [14]. The disability experienced by patients with psoriasis, for example, has been compared to that of other chronic conditions such as heart disease and diabetes [15].

HRQoL in adult dermatologic conditions is frequently assessed in studies using dermatology-specific PROMs such as the Dermatology Life Quality Index (DLQI) or Skindex (SkinDex-16 or SkinDex-29). Although both the DLQI and Skindex are validated for clinical use, Skindex-16 has been shown to be more sensitive for mild impairments in HRQoL [16]. The DLQI contains 10 questions on patients’ perception of skin diseases and impact on daily life over the past week. The Skindex-16 includes questions about symptoms (itching, burning, pain), emotional disturbances, and impact on social interactions and daily life. 

Neither the DLQI nor Skindex include questions related to cancer diagnosis and treatment, such as fear of dying or caregiver burden. Among patients with MF, the majority (80%) worry about dying from the disease [12], which is not captured by the dermatologic-specific PROMs. In contrast to cancer patients, dermatology patients including those with MF do not feel they understand their disease [17,18]. Patients report poor insight into their condition, with some believing MF was caused by anxiety. Similarly, studies of patients with alopecia have reported distress associated with illness beliefs that may not represent the actual cause of their disease [19,20].

## 4. Cancer-Specific Assessments of HRQoL

HRQoL in cancer patients can be measured using multiple instruments, including the European Organisation for the Research and Treatment of Cancer (EORTC QLQ-C30) or the Functional Assessment of Cancer Therapy-General (FACT-G). The EORTC QLQ-C30 is a 30-question tool to analyze quality of life in all cancer patients. The core questionnaire asks about exertion, activities of daily living, and symptoms over the last week including pain, insomnia, nausea, vomiting, diarrhea, and constipation. There are also shorter EORTC questionnaires for palliative cancer patients to assess functional abilities. The EORTC has developed a library of 75 questionnaires looking at quality of life in cancer patients that are available in over 120 languages. FACT-G is the core measure of the Functional Assessment of Chronic Illness Therapy (FACIT) measurement system, and is a 27-item assessment measuring physical, social, emotional, and functional well-being. 

These cancer assessments include questions about treatment side effects, emotional disturbances, social interactions, activities of daily living, and family support. Cancer-specific symptoms such as nausea and insomnia are included, but dermatologic symptoms are not.

## 5. MF/SS-Specific Assessments of HRQoL

There is currently only one published PROM that has been validated specifically for use in patients with MF/SS, the MF/SS-CTCL QoL [21]. The MF/SS-CTCL is an online only instrument developed in collaboration with the PatientsLikeMe Open Research Exchange. The MF/SS-CTCL QoL study relied on self-reported participant data, and the MF/SS-CTCL QoL score did not differ significantly by stage, suggesting a lower responsiveness when compared to other instruments.

Table 3 compares domains addressed by the dermatologic-specific and cancer-specific QOL questionnaires. 

## 6. Impact on HRQoL in Patients with MF/SS: What Is Currently Known 

MF/SS have been shown to impair multiple dimensions of HRQoL due to pruritus, physical disfigurement, treatment side effects, time and financial burdens associated with high healthcare utilization, and psychological distress [12,22,23]. The impact of MF/SS on HRQoL is thought to be at least in part related to disease stage, with greater impacts for patients with more advanced disease; however, even patients with early-stage disease can experience significant impairment [24,25,26].

### 6.1. Physical: Skin Symptoms and Pruritus 

A large study on HRQoL of over 600 patients registered in the Mycosis Fungoides Foundation found that participants were most bothered by skin erythema (94%) and symptoms impacting choice of clothing (63%) [12]. The majority (>80%) were bothered by itching, scaling, or redness. Pruritus was more common than expected, impacting 88% of respondents. In total, 62% reported their disease made them feel unattractive and 50% reported their illness interfered with their sex life. Half (50%) reported their disease interfered with daily activities. CTCL patients expressed higher levels of anxiety and depression compared to healthy controls.

More recently, a survey of 372 CTCL patients (74% with early-stage disease) found a mean itch score in this cohort on a visual analog scale of 3.2 +/− 2.8, indicating moderate pruritus [27,28]. Higher itch scores were associated with worse HRQoL on Skindex-16 and FACT-G measurements, similar to findings in previous reports [27,29,30]. Patients with CTCL reported worse HRQoL as assessed by FACT-G compared to other patients with indolent and aggressive lymphomas [27,31]. The authors found that overall HRQoL as measured by Skindex-16 and FACT-G was worse in patients with more advanced stage disease [27]. 

A systematic review of 24 studies utilizing 18 questionnaires to assess dermatology-specific, cancer-specific, and general QOL highlighted that even patients with early-stage disease had mild-to-moderate impacts on QOL, though there were greater impairments with more advanced stage disease [32]. Again, pruritus was the most frequently reported and most impactful symptom. Both patients and caregivers reported effects of the disease on activities of daily life. 

Current CTCL guidelines focus on treatment of malignancy and do not provide guidance on pruritus management or other symptomatic care for dermatologic manifestations [8].

### 6.2. Psychosocial/Mental Health

Despite having a good prognosis relative to other hematologic malignancies, patients with MF/SS still report significant psychological distress associated with their cancer diagnosis and management. In Demierre’s 2006 study, almost all participants (94%) reported feeling worried about the seriousness of their disease and 80% were worried about dying from CTCL [12]. Patients with MF/SS can also experience insomnia or depression, further worsening QoL [33].

A qualitative study found the most common symptom in CTCL patients was frustration (44%) followed by worry about progress or spread (43%) [34]. Patients also reported a high degree of embarrassment and shame (28%).

## 7. Unmet Needs in Assessing HRQoL for Patients with MF/SS

### 7.1. Disease-Specific MF/SS HRQoL Assessment Tool

An optimal assessment tool for HRQoL in MF/SS needed, particularly for clinical use. As a disease that has overlapping features of both a cancer and a skin condition, no single existing tool captures the clinical, psychological, and social burden of this disease or has been validated in this specific patient population. The majority of existing tools used to measure HRQoL are designed for research purposes rather than every day clinical use [16]. An ideal MF/SS HRQoL assessment tool for the real-world clinical setting would need to incorporate all key domains important for patients with MF/SS, including dermatologic (skin symptoms) and oncologic domains (fear of dying), while remaining more concise and targeted than existing instruments to be incorporated in clinical workflow. 

#### 7.1.1. Instrument Responsiveness

Given that treatment is largely palliative, incorporating questionnaire results into the electronic medical record to be tracked over time may help with treatment-related decision-making and tailoring each encounter to the patient’s needs. This longitudinal measurement can better inform treatment risks and benefits discussions and overall goals of care. As such, an ideal instrument needs to be responsive, showing clinically meaningful changes along with clinical impact. 

Existing instruments may not be sensitive enough to detect impact on HRQoL in early-stage disease. The literature has shown conflicting results on HRQoL in early-stage disease with some studies showing a significant negative impact [35] and others showing minimal impact [18]. Patients with no evidence of disease and those with active early-stage MF had similar Skindex-29 scores [35].

#### 7.1.2. Relationship between HRQoL and Other Disease Outcomes

Studies found that the EuroQol Five Dimensions questionnaire identifies patients with diabetes at higher risk of diabetic complications and death. A larger review of 110 studies showed that HRQoL measures correlate with mortality in both the general population and in sub-populations with health conditions [36]. The most common diagnoses included were cardiovascular disease, kidney disease, and dialysis. The relationship between HRQoL and long-term mortality was consistent; however, the relationship between short-term quality of life correlated with mortality only in diseases with a limited life expectancy, such as terminal cancer [37,38,39,40]. Most studies used Cox proportional hazards models to assess multivariate relationships. With additional studies, it is possible that similar tools may be incorporated into prognostication for patients with MF/SS.

It is not known whether impacting aspects of HRQoL might impact other outcomes for patients with MF/SS. In patients with psoriasis, psychological distress has been associated with worse responses to PUVA and adding cognitive behavior therapy to psoriasis treatment subsequently improved outcomes [41]. Whether similar interventions could improve outcomes in patients with MF/SS has not yet been explored. 

#### 7.1.3. Side Effects and Prognosis

Although treatment-related toxicities can present challenges, in some instances, they may also predict patient response to therapy. Patients treated with immune-checkpoint inhibitors such as pembrolizumab who develop immune-related adverse effects may have a better response to treatment. Patients that required immunosuppressive treatment for diarrhea secondary to immune-checkpoint inhibitors had a statistically significant overall survival benefit [42]. Similarly, the development of a mogamulizumab-associated rash (MAR) correlates with longer progression-free survival in patients with MF or SS [43]. Through tracking of treatment side effects in an objective manner, the association between response and side effects can be further characterized. With a better understanding of the possible side effects, these can help predict overall clinical course and potentially be included in future prognostication models.

### 7.2. Health Disparities in Patients with MF/SS

#### 7.2.1. Race

There is growing evidence for the interplay between health disparities and outcomes for patients with MF/SS [44,45,46]. Black/African American patients are twice as likely as white patients to develop CTCL [47]. In a retrospective study of 292 MF/SS patients, Black/African American patients had worse overall survival with an HR of 2.88 (95% CI 1.21–6.85) [48]. Erythroderma and ulceration were associated with poorer prognosis in Black/African Americans. Hypopigmentation was associated with survival in Black/African American patients but not in white patients. Nonwhite patients have worse QOL from itching even after adjusting for itch severity [49], and African American patients reported greater emotional impact as a result of their pruritus [46]. Although the etiology for these differences is not well understood, these findings suggest that patients with MF/SS may require different psychosocial support and supportive care depending on their race. A qualitative study found that due to initial misdiagnosis, Black/African American patients with MF/SS suffered from diagnostic and therapeutic delays, which may further exacerbate the psychological distress associated with diagnosis [50]. In a large analysis of 4495 cases of MF/SS from the US National Cancer Database, Black/African American race was associated with more advanced stage and decreased overall survival [51]. 

#### 7.2.2. Gender

MF is more common in men than women but little is known about how gender influences prognosis. One review of the National Cancer Database (NCDB) found that MF was less common in women and that women were more likely to present with stage I disease [52]. Both 5- and 10-year overall survival rates were higher in women (76.9% and 63.6%, respectively) than for men (70.7%; 54.2%; *p* < 0.0001). There remained a survival benefit in female patients with MF after controlling for age and disease stage. Other studies have suggested that biological differences in female patients may serve a protective role in other forms of non-Hodgkin lymphoma [53].

Despite this suggestion of better outcomes, women with CTCL report worse HRQoL compared to men, even after controlling for disease stage [54,55]. This is consistent with prior literature in dermatology that found that when compared to men, women with psoriasis [56] and atopic dermatitis [57] reported a greater impairment in HRQoL. Newly diagnosed women and patients with alopecia are particularly impacted [55].

### 7.3. Disease Beliefs and Impact on HRQoL

Beliefs surrounding a patient’s illness have been shown to have a greater impact on psychological QOL compared to disease severity in rheumatoid arthritis patients [58]; however, there is very limited research on this in patients with MF/SS. One study of 20 patients found that although early-stage MF had little impact on QOL, poor understanding of the disease was correlated with negative emotional and cognitive effects [18]. Most patients reported they believe their disease was caused by stress, similar to perceptions of patients with atopic dermatitis [18,59]. 

### 7.4. Sexual Health and Impact on HRQoL

Questions about sexual health are included on the FACT-G questionnaire; however, there is little published regarding this dimension of HRQoL. One survey found that nearly half (47%) of patients with MF/SS report that their diagnosis has a negative impact on sexual intimacy [12]. Additional investigations are needed to confirm this finding, better understand the etiology, and identify ways to mitigate this negative impairment.

### 7.5. Impact on Caregivers and Families of Patients with CTCL

Family members of patients with MF/SS report emotional distress, physical burdens, and financial burdens associated with attending medical appointments, assisting with skin care, providing support for and advocating for their loved ones [60,61]. Family members report difficulty with communication while watching a loved one suffer [60]. Additional research is needed to confirm the biggest impacts on caregiver QOL to better address their concerns.

### 7.6. Financial Toxicity and Impact on Work

There is limited existing literature regarding the financial impact of MF/SS diagnosis and treatment. The majority of patients with MF/SS (61%) feel financially burdened by their illness, and 55% of patients felt fatigue or impaired sleep, which resulted in missed school or work [12]. Financial impacts associated with MF/SS diagnosis and treatment may impact the patient, the healthcare system/insurance, or both parties. Direct financial costs may be associated with prescriptions, over-the-counter supportive care treatments, and office copays. Indirect financial costs include transportation, childcare, absence from school or work and caregiver costs. Given the wide range in disease manifestations and in treatment modalities, more research is needed on the financial burden of MF/SS.

## 8. Conclusions

Patients with MF/SS have impaired HRQoL on both dermatologic and cancer-specific questionnaires. The dermatologic-specific HRQoL measures include symptoms such as itching and burning but do not inquire about family support, coping skills, or fear of dying. Cancer-specific assessments include family support and fear of dying but do not measure some of the most bothersome symptoms including pruritus, burning, and impact on clothing choice. Given that the majority of treatments for MF/SS are palliative and focused on alleviating symptoms, a concise MF/SS HRQoL questionnaire is an unmet need for this patient population, particularly for clinical use. Development and validation of an MF/SS-specific HRQoL assessment has the potential to monitor the impact of HRQoL on treatment response and help influence medical decision-making. By incorporating this instrument into the EMR, clinically meaningful changes in HRQoL can be tracked over time and addressed promptly. Ideally instruments should be responsive across all stages, including early-stage disease.

Additional research is needed to expand on HRQoL disparities, disease beliefs/patient education, sexual wellness, impact on social/caregivers, and financial toxicity.

## Figures and Tables

**Table 1:** Staging criteria for MF/SS.

Clinical Stage	T (Skin)	N (Node)	M (Visceral)	B (Blood Involvement)
IA Limited skin involvement	T1 Patches, papules, and/or plaques on <10% BSA	N0	M0	B0-1
IB Skin-only disease	T2 Patches, papules, and/or plaques covering ≥ 10% BSA	N0	M0	B0-1
IIA	T1-2	N1-2	M0	B0-1
IIB Tumor-stage disease	T3 At least 1 tumor ≥ 1 cm in diameter	N0-2	M0	B0-1
IIIA Erythrodermic disease	T4 Confluence of erythema ≥ 80% BSA	N0-2	M0	B0 ≤5% atypical lymphocytes in peripheral blood
IIIB Erythrodermic disease	T4 Confluence of erythema ≥ 80% BSA	N0-2	M0	B1 Low tumor burden >5% atypical lymphocytes in peripheral blood
IVA_1_	T1-4	N0-2	M0	B2 High tumor burden
IVA_2_	T1-4	N3	M0	B0-2
IVB	T1-4	N0-3	M1	B0-2
Large cell transformation				

Adapted from Olsen et al. 2011 and 2022 [2,3]. BSA: body surface area; cm: centimeter.

**Table 2:** Common MF/SS treatments and adverse effects.

Systemic Treatment	Mechanism of Action	Adverse Effects
Alemtuzumab	Anti-CD52 monoclonal antibody	Injection site reactions, cytopenias
Bexarotene	Retinoid	Hypertriglyceridemia, hypothyroidism, rash, headache, neutropenia
Brentuximab vedotin	Anti-CD30 monoclonal antibody	Neuropathy, nausea, fatigue, diarrhea
Extracorporeal photopheresis	Radiation-induced apoptosis	Anemia, hypotension, thrombocytopenia
Gemcitabine	Nucleoside analog	Nausea, vomiting, flu-like symptoms
Interferon	Cytokine-induced signaling	Flu-like symptoms
Liposomal doxorubicin	Targeted stabilization of topoisomerase II within cancer cells	Lymphopenia, hand–foot syndrome
Methotrexate	Inhibits dihydrofolate reductase	Cytopenias, liver disease
Mogamulizumab	Anti-CCR4	Infusion-related reactions, diarrhea, fatigue, mogamulizumab-associated rash
Pembrolizumab	Anti-PD-1	Dermatitis, colitis, hypothyroidism
Pralatrexate	Folate analog	Mucositis, fatigue, nausea, fluid retention
Romidepsin	HDAC inhibitor	Dysguesia, nausea, fatigue, thrombocytopenia, anemia
Vorinostat	HDAC inhibitor	Diarrhea, fatigue, nausea, deep venous thrombosis

**Table 3:** Domains addressed by select QOL questionnaires in CTCL.

Domain	SkinDex	FACT-G	MF/SS-CTCL QOL
Nausea		X	
Pain		X	
Treatment side effects		X	X
Feel ill		X	
Spend time in bed		X	
Feel close to friends/interactions with others		X	X
Sexual health		X	
Feel sad or nervous		X	X
Coping with my illness		X	X
Losing hope		X	X
Worry about dying		X	
Able to work/affect daily activity		X	X
Sleeping well		X	
Itching	X		
Burning/stinging	X		
Able to enjoy activities		X	

## Abbreviations

EORTC: European Organisation for Research and Treatment of Cancer; CTCL: cutaneous T-cell lymphoma; FACT-G: Functional Assessment of Cancer Therapy—General; HRQoL: health-related quality of life; MF: mycosis fungoides; NCDB: National Cancer Database; QOL: quality of life; PROM: patient-reported outcome measure; SS: Sezary syndrome
